# Pharmacokinetic and pharmacodynamic properties of pantoprazole in calves

**DOI:** 10.3389/fvets.2022.1101461

**Published:** 2023-01-30

**Authors:** Jeff D. Olivarez, Pierre-Yves Mulon, Lisa S. Ebner, Haley Cremerius, Channing Cantrell, Rebecca Rahn, Windy Soto-Gonzalez, Joan Bergman, Sherry Cox, Jonathan P. Mochel, Amanda J. Kreuder, Joe S. Smith

**Affiliations:** ^1^Biomedical Sciences, College of Veterinary Medicine, Iowa State University, Ames, IA, United States; ^2^Large Animal Clinical Sciences, College of Veterinary Medicine, University of Tennessee, Knoxville, Knoxville, TN, United States; ^3^College of Veterinary Medicine, Lincoln Memorial University, Harrogate, TN, United States; ^4^Biomedical and Diagnostic Sciences, University of Tennessee, Knoxville, Knoxville, TN, United States; ^5^Veterinary Diagnostic and Production Animal Medicine, College of Veterinary Medicine, Iowa State University, Ames, IA, United States; ^6^Veterinary Microbiology and Preventive Medicine, College of Veterinary Medicine, Iowa State University, Ames, IA, United States

**Keywords:** bovine, ulcer, abomasum, cattle, proton pump inhibitor

## Abstract

**Introduction:**

Development of abomasal ulceration is a large concern, especially within calves; however, there is a paucity of research into the use of gastro protectants in ruminant species. Proton pump inhibitors, such as pantoprazole, are widely used in humans and companion animals. Their efficacy in ruminant species is undetermined. The objectives of this study were to 1) estimate the plasma pharmacokinetic parameters for pantoprazole in neonatal calves after three days of intravenous (IV) or subcutaneous (SC) administration, and 2) measure the effect pantoprazole had on abomasal pH over the treatment period.

**Methods:**

Pantoprazole was administered to 6 Holstein-Angus cross bull calves at a dose of 1 mg/kg (IV) or 2 mg/kg (SC), once a day (every 24 h) for three days. Plasma samples were collected over a 72 h period and analyzed *via* HPLC-UV for determining pantoprazole concentrations. Pharmacokinetic parameters were derived via non-compartmental analysis. Abomasal (n= 8) samples were collected *via* abomasal cannulas over a 12 h period, per calf per day. Abomasal pH was determined *via* a bench top pH analyzer.

**Results:**

Following Day 1 of IV administration, plasma clearance, elimination half-life, and volume of distribution of pantoprazole were estimated at 199.9 mL/kg/h, 1.44 h, and 0.51 L/kg, respectively. On Day 3 of IV administration, the reported values were 192.9 mL/kg/h, 2.52 h, and 1.80 L/kg mL, respectively. Elimination half-life and volume of distribution (V/F) of pantoprazole following SC administration were estimated at 1.81 h and 0.55 L/kg, respectively, on Day 1; and 2.99 h and 2.82 L/kg, respectively, on Day 3.

**Discussion:**

The reported values for IV administration were similar to those previously reported in calves. SC administration appears to be well absorbed and tolerated. The sulfone metabolite was detectable for 36 h after the last administration for both routes. Abomasal pH was significantly higher than the pre-pantoprazole pH 4, 6, and 8 h after administration in both the IV and SC groups. Further studies of pantoprazole as a treatment/preventative for abomasal ulcers are warranted.

## 1. Introduction

Ulceration of the gastrointestinal system is a complex disease that has been documented in many domesticated species (1–5). Specifically, in cattle, abomasal ulceration is a common cause of morbidity and mortality throughout the beef and dairy industries (1, 6). Identified factors contributing to ulceration include stress (e.g., handling, travel), weather, housing, mineral deficiencies, bacterial overgrowth, and the use of non-steroidal anti-inflammatory drugs (NSAIDs) (7). The animal's age may also contribute to the disorder, with the highest reported prevalence in veal calves; however, ulcers can be found in all ages of cattle (1, 8). Production of hydrochloric acid (HCl) by gastric parietal cells has been proven to exacerbate ulceration and is believed to contribute to the establishment of ulcers (9). Many pharmaceutical therapies for gastric ulceration in non-ruminant species are directed toward the reduction of gastric HCl because of its role in ulcer formation (4, 5, 10). It is proposed that reducing HCl is therapeutic for ruminants, although the complete etiology of abomasal ulcerations is unknown.

Pantoprazole is a proton pump inhibitor that irreversibly binds to the hydrogen potassium ATPase pumps on the luminal surface of parietal cells in the gastric mucosa to prevent acid secretion (11). Pantoprazole is labeled for the treatment of peptic ulcers in humans by reducing acid secretion and increasing gastric pH (12). Increasing gastric pH to >4 for >66% of a 24 h period has been the suggested therapeutic window in promoting the healing of gastric ulcers in humans and horses (13). Studies in both foals and alpacas have shown that intravenous pantoprazole effectively increases gastric pH, but studies in ruminant species are lacking (14, 15). Several retrospective investigations demonstrate the use of pantoprazole in clinical bovine patients, but no pharmacodynamic information exists (16–18). A recent study investigating the pharmacokinetic properties of IV pantoprazole in neonatal calves demonstrated a longer elimination half-life of pantoprazole in calves and similar clearance compared to both alpacas and foals, suggesting IV pantoprazole may be an effective therapeutic option in ruminants if it possesses similar pharmacodynamic properties (19). The study of pantoprazole in alpacas also indicated subcutaneous administration of pantoprazole effectively reduced third compartment (C3) gastric acid secretion (14). There are currently no studies investigating the use of subcutaneous pantoprazole in ruminants; however, intravenous access is not always manageable in cattle, and there is a need for alternative parenteral routes.

The primary objectives of this study were to determine the pharmacokinetic and pharmacodynamic properties of pantoprazole after multi-day intravenous (IV) and subcutaneous (SC) administration of pantoprazole in neonatal calves as well as to report the pharmacokinetics of the sulfone metabolite in these calves.

## 2. Materials and methods

### 2.1. Animals

This study was completed at the University of Tennessee's Veterinary Research and Education Center. Six Holstein-Angus bull calves procured from a single source farm were enrolled in the study. The age of these calves at enrollment was 17–18 days, with an average weight of 55.8 ± 4.6 kg. The study was approved by the Institution Animal Care and Use Committee (IACUC # 2825-05221) at the University of Tennessee. The calves were individually housed in hutches under a shaded pavilion. Enrollment criteria for this study included no previous medical history of illness, no previous administration of medication, as well as a physical examination by a veterinarian that yielded normal vital parameters for a bovine calf. Vaccination status was unknown for all calves. All calves received a diet of commercial non-medicated milk replacer that either met or exceeded the National Research Council (NRC) requirements for the maintenance and growth of bovine calves. All calves had *ad libitum* access to water.

Two weeks prior to enrollment in the study, the calves underwent a procedure to place abomasal cannulas (Bard Gastrostomy Buttons; BB) as previously described (20). Briefly, the calves were anesthetized using a total intravenous anesthesia (TIVA) protocol of xylazine (0.1 mg/kg), butorphanol (0.1 mg/kg), and ketamine (1 mg/kg boluses, as needed). Immediately prior to surgery, the calves were administered a single dose of flunixin meglumine (1.1 mg/kg, IV) and a single dose of procaine penicillin (22,000 IU/kg, IM). Once anesthetized, the calves were placed in left lateral recumbency. The right paracostal area was clipped and aseptically prepped using povidine surgical scrub and 70% alcohol. The skin was then infused with 10–15 mLs of 2% lidocaine in an “inverted-L” pattern, craniodorsal to the incision. A 5 cm longitudinal incision was then made through the skin and underlying muscle layers, starting 3 cm caudal to the rib cage and ~10 cm lateral to ventral midline. The body of the abomasum was then identified and pexied to the body wall using two horizontal mattress sutures at the cranial and caudal portions of the body wall incision. The cannulas were then inserted *via* a stab incision through the body wall and into the abomasum and secured with a purse-string suture. The calves were then recovered, and the surgical sites were allowed to heal for 14 days prior to study initiation.

### 2.2. Experimental design

The six calves were randomly allocated (by coin toss) into either one of two groups: intravenous (IVG) or subcutaneous (SCG) administration groups.

Surgical implantation of all six calves was completed over a 2 day period, and all animals were given at least 14 days to recover prior to the start of the experiment. Abomasal contents were collected for a 12 h period before pantoprazole administration (day 0), at time points 0, 1, 2, 3, 4, 6, 8, and 12 h.

On day 1, 1 h before the initiation of the study, the calves were restrained with a rope halter, and an IV jugular catheter was placed aseptically in all calves. Preceding catheter placement, the skin was aseptically prepared utilizing four alternating wipes of povidine surgical scrub and 70% isopropyl alcohol. The skin was then infiltrated with 1–2 mLs of 2% lidocaine, and a #15 blade was used to create a cut-down incision. A 14-gauge catheter was placed through the cut down into the jugular, and the catheter was secured to the skin using 2 Ethilon suture. A second IV catheter was then placed in the contralateral jugular vein in the IVG calves using the same process described above.

Pantoprazole sodium (West-Ward, Eatontown, NJ, United States) was reconstituted to a 4 mg/mL concentration using 10 mL of a 0.9% saline 1L IV bag per manufacturer's recommendations. The calves were fed ~2 h prior to T0 and at 2 h prior to T12. At T0, the IVG calves received a 1 mg/kg dose in the left jugular vein over 1 min, while the SCG calves were administered a 2 mg/kg dose subcutaneously in the neck. SC doses >10 mL were administered in more than one location, per Beef Quality Assurance (BQA) guidelines. Pantoprazole administration for the IVG and SCG calves was repeated at 24 h intervals on days 2 and 3.

On days 1 and 3 of the study, blood samples were collected at 0, 15, 30, 45, and 60 min, as well as 2, 3, 4, 8, 12, 18, and 24 h after drug administration. Samples were obtained from all calves through an IV catheter in the right jugular vein utilizing a previously described push-pull technique (21). The blood samples were immediately placed into sodium heparin tubes, then placed on ice until centrifuged at 1,500 × g for 10 min. All blood samples were processed within 6 h of collection. After centrifugation plasma was transferred to cryovials which were then stored at −80°C until analysis. Abomasal contents were collected each day of the study at 0, 1, 2, 3, 4, 6, 8, and 12 h after pantoprazole administration and processed as described below.

After a 10 day washout period, calves originally in the IVG were switched to the SCG and vice versa. The study was then repeated as described above.

### 2.3. Abomasal content collection and pH measurement

Abomasal contents were collected *via* the surgically placed BB tube. Briefly, a 3 × 70 mm stainless steel two-eyed teat cannula attached to a 12 mL syringe was introduced into the BB tube ~30 mm in order to bypass the one-way valve within the tube. Negative pressure was then gently applied using the 12 mL syringe until 4–5 mL of abomasal contents were acquired. The pH of each sample was then recorded within 15 min of collection in a process described below.

The aliquots of abomasal content were placed into a 30 mL falcon tube. A benchtop pH analyzer (UB-10 pH/mV meter, Denver Instruments, US) was then used to measure pH. The analyzer was calibrated prior to each sample set according to the manufacturer's procedure. Once calibrated, the probe was introduced into the abomasal content sample and allowed to equilibrate for 30 s, at which time the pH was recorded.

### 2.4. Analytical method

Plasma pantoprazole analysis was performed using a reverse phase high performance liquid chromatography (HPLC) method as previously described for pantoprazole and the pantoprazole sulfone metabolite in goat plasma (22). The system consisted of a 2,695 separations module and a 2,487 UV absorbance detector (Waters). The compounds were separated on a Symmetry C18 (4.6 × 150 mm, 5 μm) column with a 5 μm Symmetry C18 guard column. The mobile phase was a mixture of 0.1 M sodium phosphate dibasic and acetonitrile (68:32). The flow rate was 1 mL/min, and absorbance was measured at 290 nm.

Pantoprazole and its metabolite were extracted from plasma samples using a liquid-liquid extraction method. Previously frozen samples were thawed, vortex-mixed, and 100 μl of plasma was transferred to a 13 × 100 mm screw top tube, followed by 10 μl of tinidazole (internal standard, 100 μg/mL) and 2 mL chloroform. The tubes were rocked for 15 min and then centrifuged for 20 min at 1,000 × g. The organic layer was transferred to a glass tube and evaporated to dryness with nitrogen gas. Samples were reconstituted in 250 μL of mobile phase, and 100 μL was analyzed.

Standard curves for the plasma analysis were prepared by fortifying untreated, pooled plasma with pantoprazole and the metabolite, which produced a linear concentration range of 0.01–100 μg/mL. The average recovery for pantoprazole and it's metabolite was 100 and 90%. The average recovery for the internal standard was 99%. The quality control (QC) samples used for validation were 0.03, 0.3, 3, and 30 μg/mL, and the intra and inter-assay variability ranged from 2 to 11% for pantoprazole and 3% to 9% for the metabolite. The lower limit of quantification for both was 0.1 μg/mL.

### 2.5. Pharmacokinetic analysis

Pharmacokinetic parameters were calculated from time-plasma concentration data as previously described (19). Pharmacokinetic modeling was performed *via* standard industry modeling software (PKanalix, Monolix Suite 2021R2, Lixoft, France) as described for pantoprazole in goats and calves (19, 23). Standard data representing time vs. concentration information for pantoprazole was determined *via* HPLC from the samples collected at 8 time points ranging from 0 to 12 h after Day 1 and 3 of administration. Standard PK parameters were generated for individual calves, as follows: Maximum concentration of pantoprazole extrapolated to time zero, C0; Time of maximum pantoprazole concentration, Tmax; Area under pantoprazole concentration–time curve, AUClast and AUCinf; Area under the moment curve, AUMCinf; Pantoprazole mean residence time, MRT = AUMCinf/AUCinf; Pantoprazole terminal half-life, T_1/2_ (λz) = ln (2)/λz; Pantoprazole systemic clearance, CL = Dose/AUCinf; Volume of distribution of pantoprazole (area), Vz.

A linear/log trapezoidal rule was used for data analysis to estimate the area under the pantoprazole time-curve. Summary statistics on the individual PK parameters were performed thereafter to derive the geometric mean, median, minimum, and maximum.

Bioavailability (F) was calculated utilizing the following equation:


$$\begin{array}{l} F=\frac{AUC\left(SC\right)}{AUC\left(IV\right)}\;x\;\frac{Dose\left(IV\right)}{Dose\left(SC\right)} \end{array}$$

### 2.6. Statistical analysis

Data from pH testing was evaluated for normality. Then one-way ANOVA followed by multiple comparisons (based on distribution) testing was performed using GraphPad Prism (version 8.0.0, GraphPad Software, San Diego, CA). Day zero was the baseline being compared to individual treatment days. Significance was set at a value of *P* < 0.05.

## 3. Results

### 3.1. Animals

At the time of enrollment, all calves had normal vital parameters and were considered healthy. Surgical implantation of the BB was well-tolerated by most of the calves, with four of the six cannulas remaining in place throughout the entire study period and only mild swelling noted at the insertion site. One BB came dislodged after day 1 of the first trial and another become dislodged immediately prior to the beginning of the first trial. These calves were kept in the study for pharmacokinetic data and was included in the days 0 and 1 abomasal pH data. One of the calves developed a catheter site infection and thrombophlebitis at the IV catheter sites. Intravenous catheterization was well-tolerated by all other calves. No clinical manifestations of adverse reactions were observed due to either intravenous or subcutaneous administration of pantoprazole.

### 3.2. Pharmacokinetics

**Figure 1:**
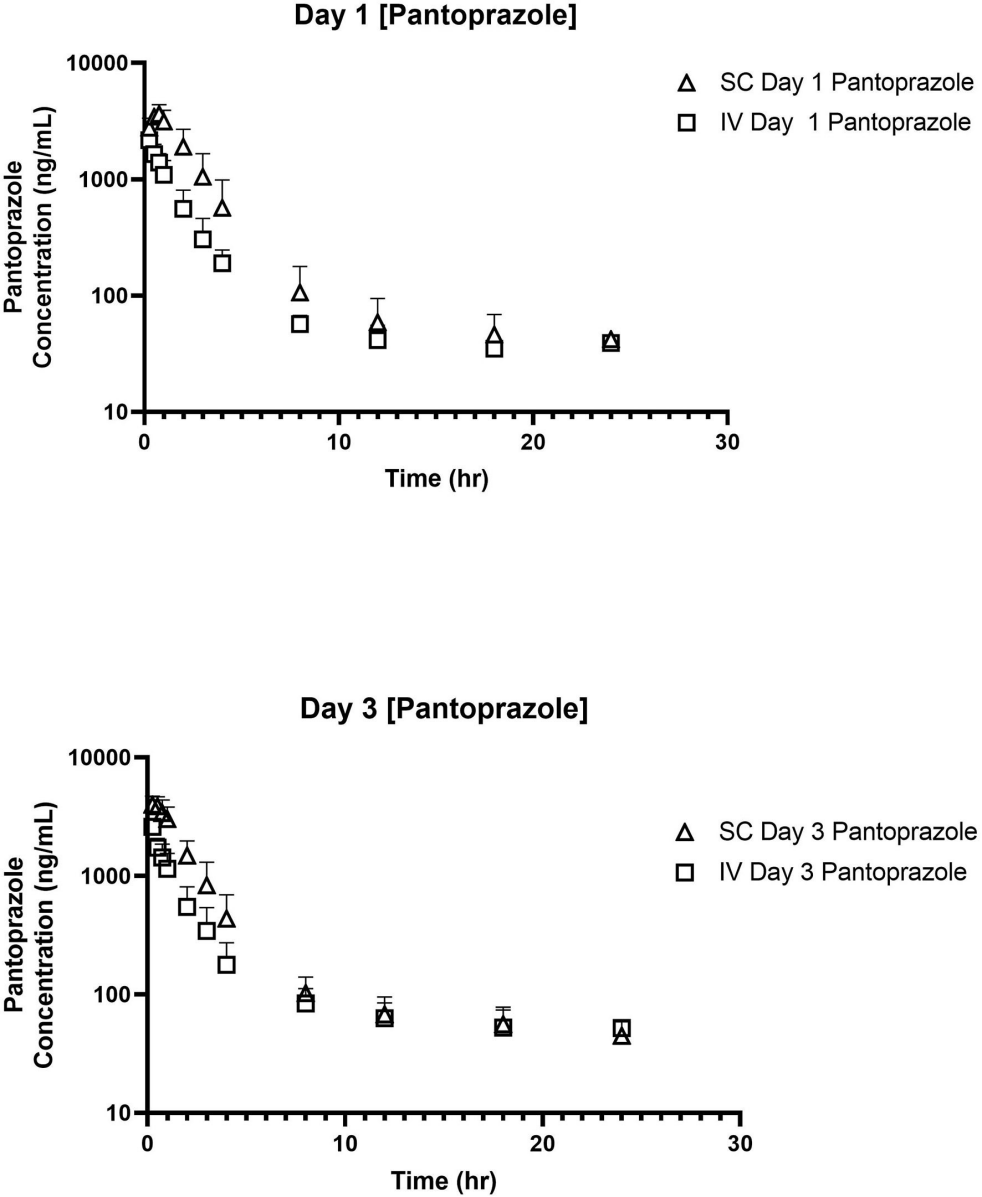
Time vs. Concentration curves for intravenous (IV; square) and subcutaneous (SC; triangle) administration of pantoprazole on Days 1 **(top)** and 3 **(bottom)**.

No concentrations of pantoprazole were detected in any of the calves prior to administration on Day 1. Table 1 displays the geometric mean, median, minimum, and maximum of the pharmacokinetic parameters of pantoprazole in calves after IV and SC administration. IV and SC administration had fairly rapid elimination with half-lives of 1.44 and 1.81 h, respectively. Table 2 displays the geometric mean, median, minimum, and maximum of the pharmacokinetic parameters of pantoprazole in calves after three consecutive days of IV and SC administration. The elimination half-lives of intravenous and subcutaneous administration had increased to 2.52 and 2.99 h, respectively. Figure 1 displays the time vs. concentration curves for pantoprazole on day 1 and 3.

**Table 1:** Day 1 pharmacokinetic parameters of pantoprazole after intravenous (IV) and subcutaneous (SC) administration in calves.

**Compound (route)**	**Parameter**	**Unit**	**Mean**	**Standard deviation**	**Median**	**Min**	**Max**
Pantoprazole (IV)	C_0_	ng/mL	2,147	257	2,190	1,815	2,519
	AUC_last_	ng/mL^*^h	3,422	1,020	4,008	1,878	4,351
	AUC_inf_	ng/mL^*^h	3,586	1,100	4,089	1,945	4,632
	AUMC_inf_	ng/mL^*^h	5,372	4,568	6,774	1,207	13,881
	MRT_inf_	h	1.95	1.2	2.08	0.76	4.1
	Cl	mL/h/kg	199.9	89.7	175.3	154.8	368.6
	λz	1/h	0.34	0.37	0.31	0.13	1.02
	T_1/2_ (λz)	h	1.44	1.29	2.25	0.68	3.9
	V_z_	L/kg	0.51	0.27	0.57	0.24	0.88
Pantoprazole (SC)	C_max_	ng/mL	3,435	771	3,436	2,350	4,553
	T_max_	h	0.58	0.21	0.75	0.25	0.75
	AUClast	ng/mL^*^h	7,629	3,832	7,070	4,270	14,646
	AUC_inf_	ng/mL^*^h	7,857	3,849	7,402	4,502	14,894
	AUMC_inf_	ng/mL^*^h	21,046	20,818	18,800	12,014	66,608
	MRT_inf_	h	2.67	1.45	2.22	1.7	5.11
	λz	1/h	0.34	0.21	0.38	0.17	0.75
	T_1/2_ (λz)	h	1.81	1.37	1.86	0.92	4.13
	V_z_/F	L/kg	0.55	0.61	0.60	0.22	1.90

C_0_, calculated concentration at time zero of IV administration; AUC_last_, area under the curve calculated at the last time point; AUC_inf_, area under the curve extrapolated to infinity; AUMC_inf_, area under the moments curve extrapolated to infinity; MRT_inf_, mean residence time extrapolated to infinity; Cl, plasma clearance; T_1/2_ (λz), elimination half-life [reported as harmonic mean (34, 35)]; V_z_, volume of distribution; C_max_, maximum plasma concentration after SC administration; T_max_, time to reach maximum plasma concentration after SC administration; V_z_/F, volume of distribution accounting for bioavailability; λz, elimination rate constant. Means reported as Geometric mean, with the exception of elimination half-life, which is reported as harmonic mean.

**Table 2:** Day 3 pharmacokinetic parameters of pantoprazole after intravenous (IV) and subcutaneous (SC) administration in calves.

**Compound (route)**	**Parameter**	**Unit**	**Mean**	**Standard deviation**	**Median**	**Min**	**Max**
Pantoprazole (IV)	C_0_	ng/mL	2,575	371	2,592	2,105	3,214
	AUC_last_	ng/mL^*^h	4,580	1,123	4,635	2,933	5,899
	AUC_inf_	ng/mL^*^h	5,171	994	5,257	3,500	6,212
	AUMC_inf_	ng/mL^*^h	27,338	21,737	24,098	12,058	68,709
	MRT_inf_	h	5.29	4.11	5.22	2.36	12.91
	Cl	mL/h/kg	192.9	36.5	188.1	158.1	255.0
	λz	1/h	0.11	0.13	0.078	0.047	0.35
	T_1/2_ (λz)	h	2.52	1.86	2.71	1.99	6.91
	V_z_	L/kg	1.80	1.96	2.65	0.54	4.67
Pantoprazole (SC)	C_max_	ng/mL	4,221	604	4,326	3,439	5,085
	T_max_	h	0.41	0.21	0.5	0.25	0.75
	AUClast	ng/mL^*^h	8,797	2,617	9,289	5,010	11,512
	AUC_inf_	ng/mL^*^h	9,417	2,789	9,838	5,585	12,336
	AUMC_inf_	ng/mL^*^h	45,031	26,684	47,165	20,159	84,666
	MRT_inf_	h	4.78	1.75	5.79	2.26	6.86
	λz	1/h	0.077	0.094	0.057	0.053	0.27
	T_1/2_ (λz)	h	2.99	0.87	3.63	1.76	3.79
	V_z_/F	L/kg	2.82	2.06	3.08	0.79	6.52

C_0_, calculated concentration at time zero of IV administration; AUC_last_, area under the curve calculated at the last time point; AUC_inf_, area under the curve extrapolated to infinity; AUMC_inf_, area under the moments curve extrapolated to infinity; MRT_inf_, mean residence time extrapolated to infinity; Cl, plasma clearance; T_1/2_ (λz), elimination half-life; V_z_, volume of distribution, C_max_, maximum plasma concentration after SC administration; T_max_, time to reach maximum plasma concentration after SC administration; V_z_/F, volume of distribution accounting for bioavailability; λz, elimination rate constant. Means reported as Geometric mean, with the exception of elimination half-life, which is reported as harmonic mean.

**Figure 2:**
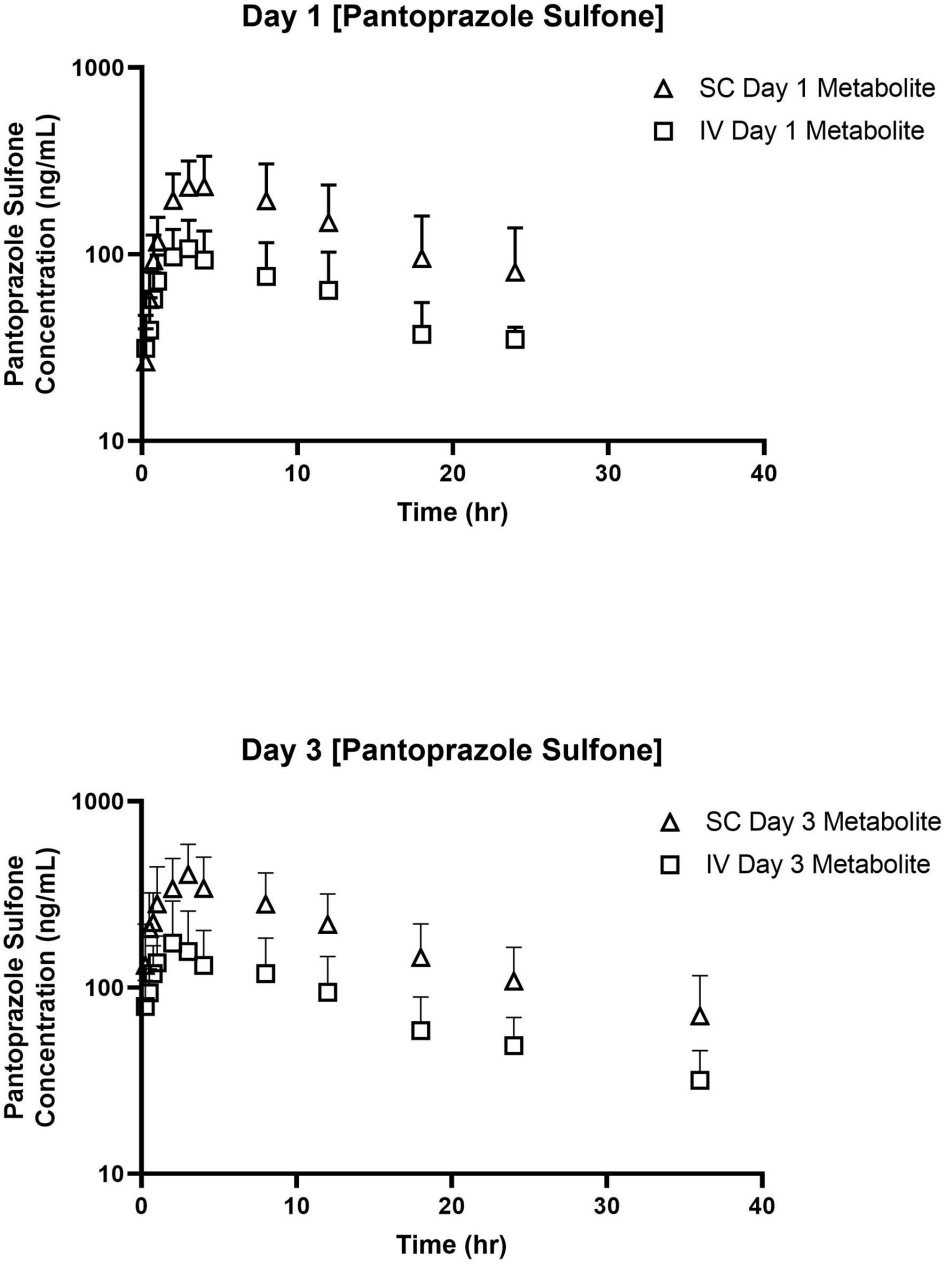
Time vs. Concentration curves for pantoprazole sulfone after intravenous (IV; square) and subcutaneous (SC; triangle) administration of pantoprazole on Days 1 **(top)** and 3 **(bottom)**.

Tables 3, 4 display the geometric mean, median, minimum, and maximum of the pharmacokinetic parameters of the sulfone metabolite on days 1 and 3, respectively. Figure 2 displays the time vs. concentration curves for the sulfone metabolite on days 1 and 3.

**Table 3:** Day 1 pharmacokinetic parameters of pantoprazole sulfone after intravenous (IV) and subcutaneous (SC) administration of pantoprazole sodium in calves.

**Compound (route)**	**Parameter**	**Unit**	**Mean**	**Standard deviation**	**Median**	**Min**	**Max**
Pantoprazole sulfone (IV)	C_max_	ng/mL	105	41	103	60	162
	T_max_	h	2.45	1.03	3	1	4
	AUC_last_	ng/mL^*^h	1,131	707	1,355	407	2,415
	AUC_inf_	ng/mL^*^h	1,472	861	1,749	647	2,867
	AUMC_inf_	ng/mL^*^h	18,954	15,328	26,653	67,867	39,042
	MRT_inf_	h	12.85	3.57	12.89	8.46	18.49
	λz	1/h	0.084	0.024	0.085	0.058	0.12
	T_1/2_ (λz)	h	8.0	2.21	8.17	5.92	11.9
Pantoprazole sulfone (SC)	C_max_	ng/mL	231	91	213	138	351
	T_max_	h	3.57	0.55	4	3	4
	AUClast	ng/mL^*^h	2,908	1,857	3,188	1,146	5,546
	AUC_inf_	ng/mL^*^h	3,657	3,006	4,576	1,273	8,767
	AUMC_inf_	ng/mL^*^h	50,251	77,571	84,165	10,330	203,632
	MRT_inf_	h	13.74	6.84	14.2	8.12	23.23
	λz	1/h	0.085	0.053	0.08	0.048	0.17
	T_1/2_ (λz)	h	7.2	4.62	8.7	4.12	14.4

AUC_inf_, area under the curve extrapolated to infinity; AUMC_inf_, area under the moments curve extrapolated to infinity; MRT_inf_, mean residence time extrapolated to infinity; T_1/2_ (λz), elimination half-life; C_max_, maximum plasma concentration; T_max_, time to reach maximum plasma concentration; λz, elimination rate constant. Means reported as Geometric mean, with the exception of elimination half-life, which is reported as harmonic mean.

**Table 4:** Day 3 pharmacokinetic parameters of pantoprazole sulfone after intravenous (IV) and subcutaneous (SC) administration of pantoprazole sodium in calves.

**Compound (route)**	**Parameter**	**Unit**	**Mean**	**Standard deviation**	**Median**	**Min**	**Max**
Pantoprazole sulfone (IV)	C_max_	ng/mL	153	116	147	63	401
	T_max_	h	2.14	1.03	2	1	4
	AUC_last_	ng/mL^*^h	2,273	1,340	2,611	678	4,527
	AUC_inf_	ng/mL^*^h	2,743	1,661	3,434	780	4,968
	AUMC_inf_	ng/mL^*^h	47,460	47,852	60,600	7,438	123,356
	MRT_inf_	h	17.34	7.66	16.68	9.53	29.03
	λz	1/h	0.061	0.03	0.064	0.035	0.12
	T_1/2_ (λz)	h	10.5	5.39	10.98	5.92	19.66
Pantoprazole sulfone (SC)	C_max_	ng/mL	383	181	326	272	712
	T_max_	h	2.77	0.45	3	2	3
	AUClast	ng/mL^*^h	5,834	3,048	5,312	3,288	10,870
	AUC_inf_	ng/mL^*^h	7,086	4,321	6,210	3,708	13,924
	AUMC_inf_	ng/mL^*^h	141,436	125,690	117,261	58,146	325,838
	MRT_inf_	h	19.96	4.77	18.88	15.68	27.09
	λz	1/h	0.051	0.013	0.055	0.039	0.067
	T_1/2_ (λz)	h	12.8	3.49	12.7	10.4	17.94

AUC_inf_, area under the curve extrapolated to infinity; AUMC_inf_, area under the moments curve extrapolated to infinity; MRT_inf_, mean residence time extrapolated to infinity; T_1/2_ (λz), elimination half-life; C_max_, maximum plasma concentration; T_max_, time to reach maximum plasma concentration; λz, elimination rate constant. Means reported as Geometric mean, with the exception of elimination half-life, which is reported as harmonic mean.

Bioavailability of the first SC administration was 115.2 ± 56.0 %.

### 3.3. pH investigation

**Figure 3:**
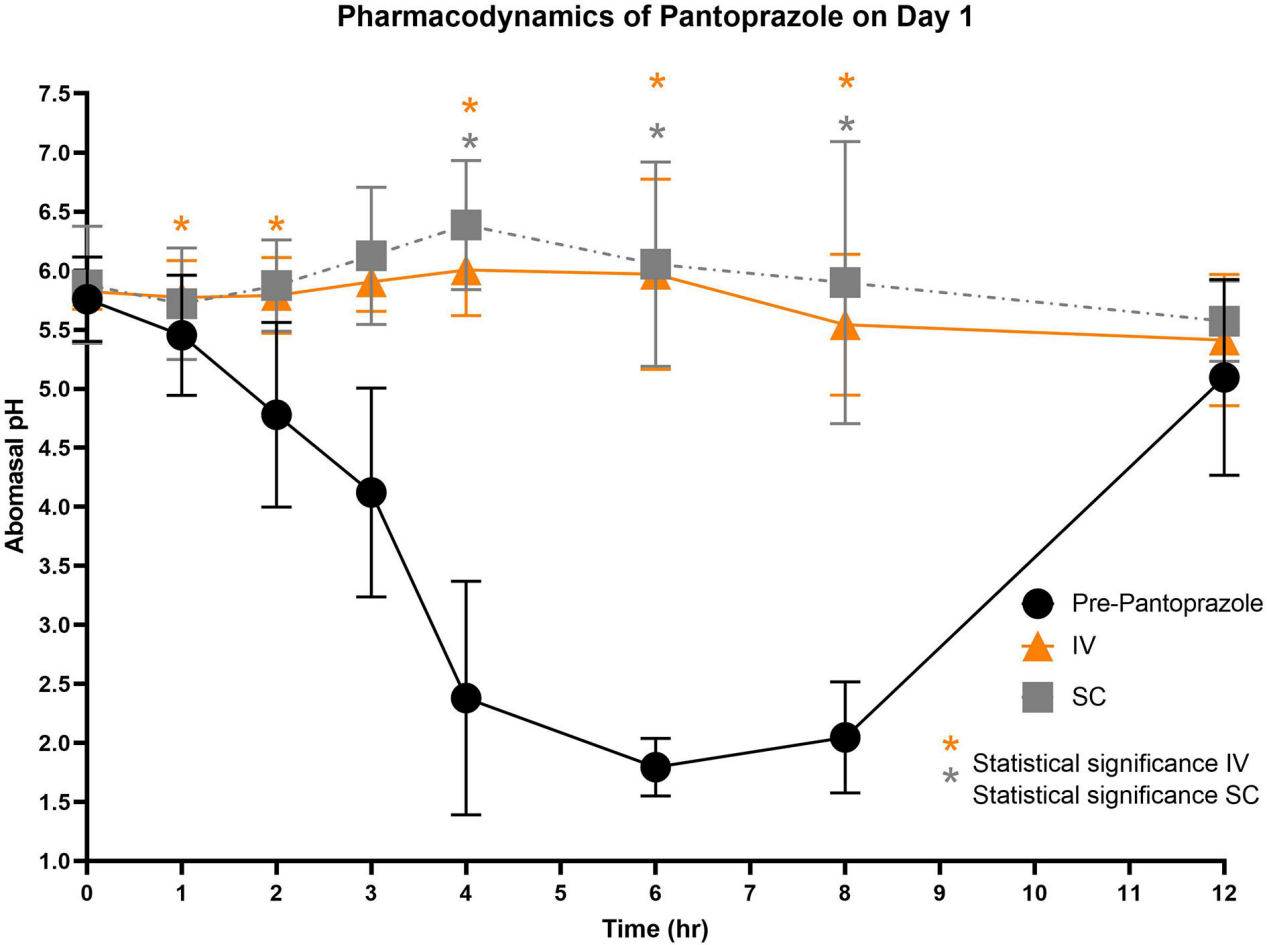
Comparison of abomasal pH over time between control, IV, and SC treatment groups on Day 1. Black circles, pH pre-pantoprazole administration; Orange triangles, pH after IV pantoprazole administration; Gray square, pH after SC administration. Statistical significance (*P* < 0.05) noted with asterisk.

**Figure 4:**
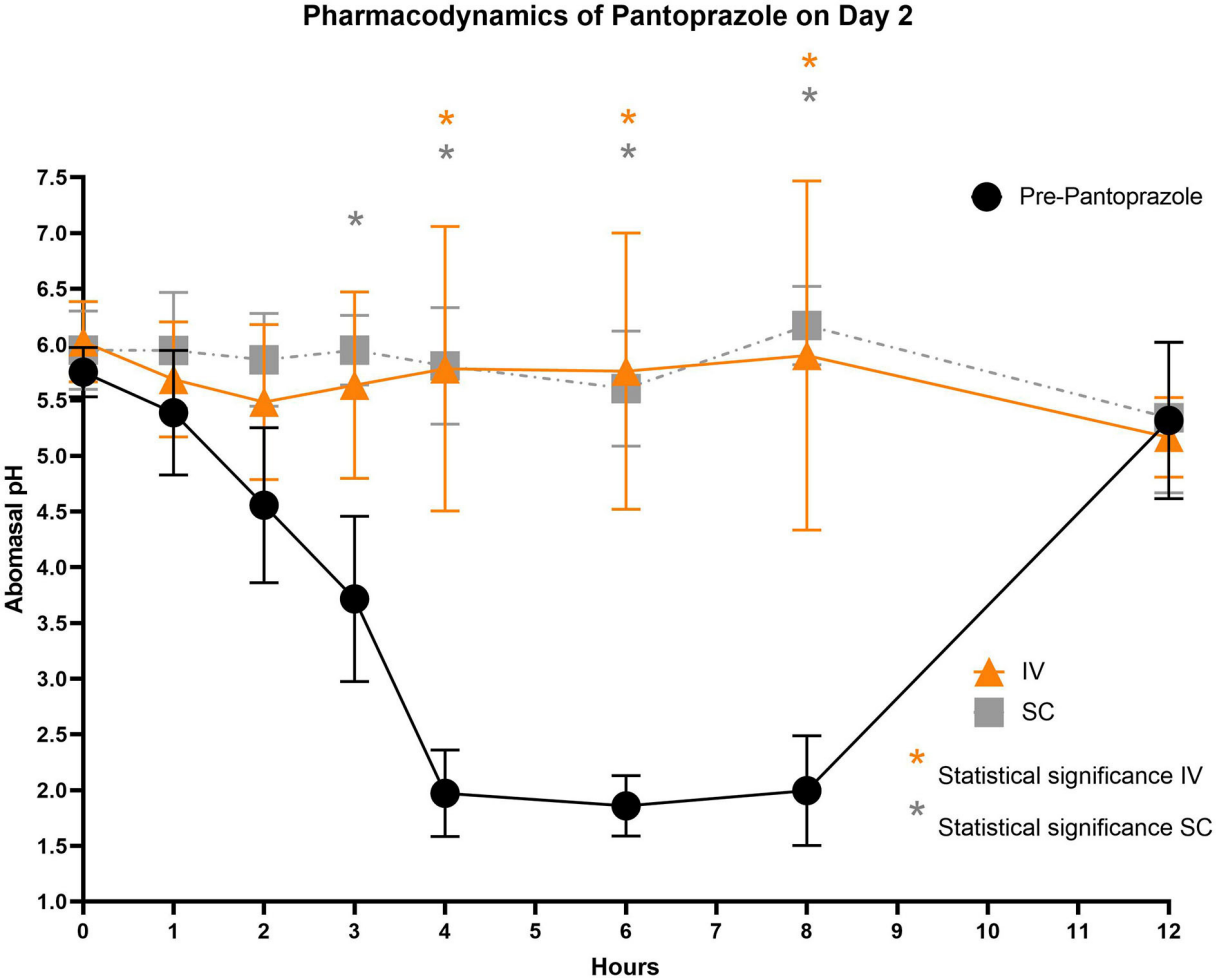
Comparison of abomasal pH over time between control, IV, and SC treatment groups on Day 2. Black circles, pH pre-pantoprazole administration; Orange triangles, pH after IV pantoprazole administration; Gray square, pH after SC administration. Statistical significance (*P* < 0.05) noted with asterisk.

**Figure 5:**
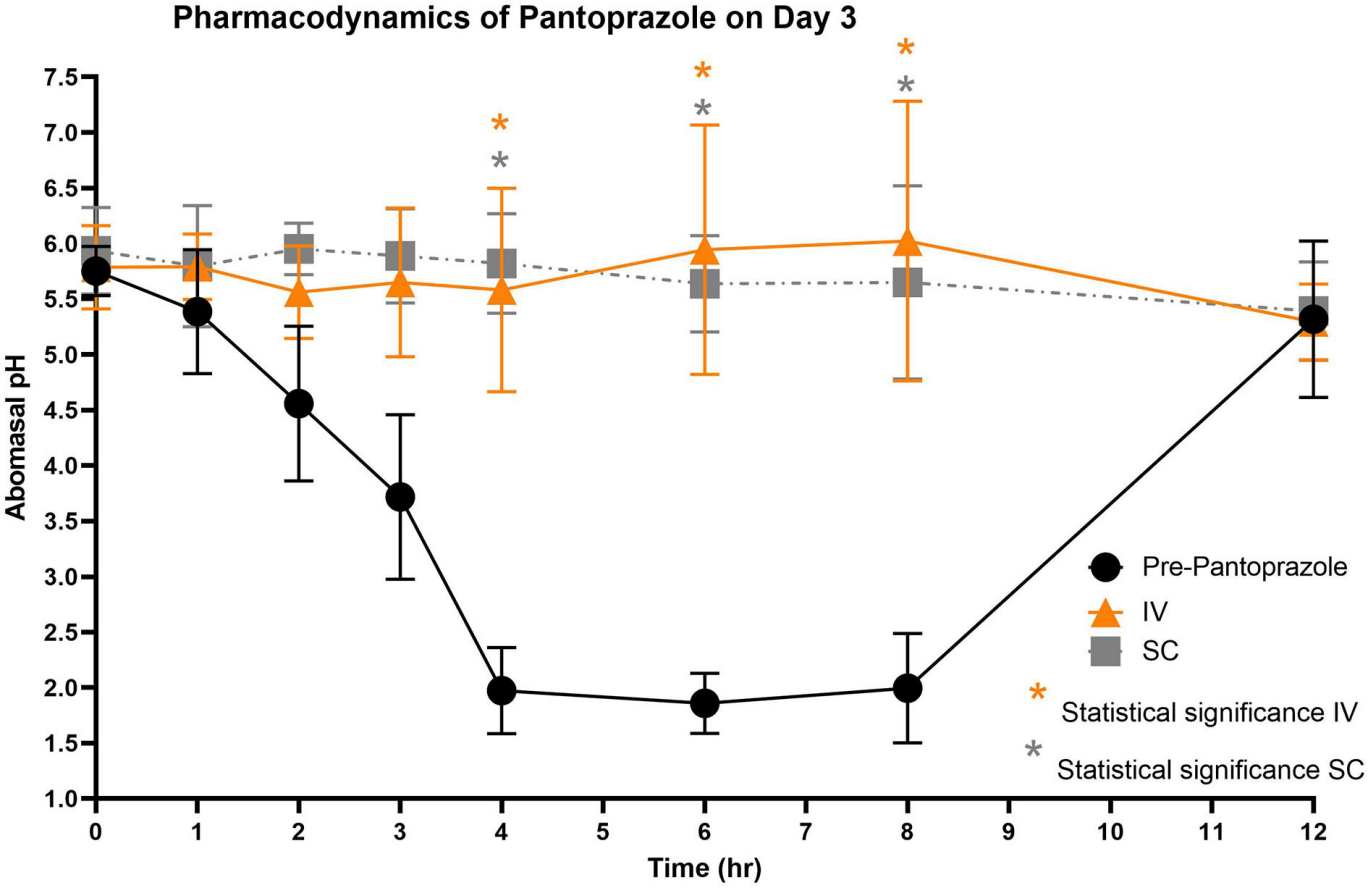
Comparison of abomasal pH over time between control, IV, and SC treatment groups on Day 3. Black circles, pH pre-pantoprazole administration; Orange triangles, pH after IV pantoprazole administration; Gray square, pH after SC administration. Statistical significance (*P* < 0.05) noted with asterisk.

Figures 3–5 compare the abomasal pH between the control and treatment groups on days 1–3, respectively. Of note, both treatment groups had a significantly higher pH at the 4, 6, and 8 h time points compared to the control pretreatment pH on all 3 days. Tables 5, 6 display the average pH at each time point for IV and SC pantoprazole, respectively.

**Table 5:** Comparison of average hourly abomasal pH after intravenous administration of pantoprazole.

**Time (h)**	**Day 0**	**Day 1**	**Day 2**	**Day 3**
0	5.778	5.912	6.025	5.785
1	5.366	5.754	5.6875	5.79
2	4.546	5.872	5.4825	5.5625
3	3.972	6.264	5.635	5.65
4	2.452	6.54^c^	5.7825^a^	5.5875^b^
6	1.86	6.35^c^	5.76^b^	5.945^b^
8	2.018	6.196^a^	5.9025^a^	6.0225^a^
12	5.334	6.286	5.1675	5.295

a Indicates statistically significant difference (p-value of 0.05–0.01) from the control (Day 0).

b Indicates statistically significant difference (p-value of 0.01–0.005) from the control (Day 0).

c Indicates statistically significant difference (p-value of < 0.005) from the control (Day 0).

**Table 6:** Comparison of average hourly abomasal pH after subcutaneous administration of pantoprazole.

**Time (h)**	**Day 0**	**Day 1**	**Day 2**	**Day 3**
0	5.765	5.825	5.95	5.935
1	5.5175	5.7725	5.9425	5.795
2	5.0025	5.79	5.8625	5.9525
3	4.5275	5.905	5.95	5.8875
4	2.79	6.005^a^	5.8075^a^	5.82^a^
6	1.73	5.97^c^	5.605^c^	5.6375^c^
8	2.1	5.5425^c^	6.17^c^	5.65^b^
12	4.8725	5.4125	5.3425	5.3925

a Indicates statistically significant difference (p-value of 0.05–0.01) from the control (Day 0).

b Indicates statistically significant difference (p-value of 0.01–0.005) from the control (Day 0).

c Indicates statistically significant difference (p-value of < 0.005) from the control (Day 0).

## 4. Discussion

To our knowledge, this is the first report investigating the pharmacokinetic and pharmacodynamic properties of multi-day intravenous and subcutaneous administration of pantoprazole in calves. In this study, both IV and SC administration of pantoprazole maintained abomasal pH to > 4 in calves over a 12 h period.

The pharmacokinetic properties of pantoprazole after 1 day of IV administration were less than those previously reported (19). The half-life of pantoprazole reported in that study was 2.81 h, while our current study found a half-life of 1.44 h. Differences between these values could be due to variations between measurement techniques as well as differences in lower limits of quantifications (LLOQ). The LLOQ for the original pharmacokinetic study was reported to be much lower than our current study's (0.002 and 0.1μg/mL, respectively). The ability to detect smaller amounts of pantoprazole in the plasma could account for the longer half-life (24). For subcutaneous administration, the Cmax (3,434.77 ng/mL) was achieved fairly rapidly (within 0.58 h), indicating the pantoprazole was rapidly absorbed, which is also reported in human studies (25).

Interestingly the pharmacokinetic data for day 3 demonstrated potential accumulation of the drug with multiple-day administration, as Cmax, AUC, MRT, and T_1/2_ had all increased for both IV and SC groups. However, due to the short elimination half-lives, this could be non-linear kinetics in disposition, possibly due to enzymatic inhibition. Pantoprazole is rapidly metabolized by the liver's cytochrome P450 (CYP) system (26). Previous studies have indicated that some PPIs [such as omeprazole (27)] can inhibit the CYP, reducing its efficacy in breaking down the drug into its metabolites, meaning the parent drug can be detected in higher concentrations after repeat administrations; however, pantoprazole has not been shown to inhibit the CYP in humans (28). If this is true in ruminants, then increases in the pharmacokinetic values on Day 3 may be due to the saturation of the CYP mechanism rather than inhibition. Despite increased pantoprazole concentrations in the plasma, there was no significant difference in abomasal pH between Day 1 and Day 3 of treatment.

On all 3 days of testing, the abomasal pH was significantly higher than the pre-pantoprazole pH 4, 6, and 8 h after administration in both the IVG and SCG. As seen in Figures 3–5, the pretreatment pH steadily declines during the first 4 h of the study, then maintains a pH of around 2.0 from the 4–8 h period. The calves were fed a commercial milk replacer ~2 h before the 0 h and the 12 h time points. The pH of the milk replacer was ~6.61 ± 0.046, which may account for the relatively high abomasal content pH at 0 h and the drastic increase in pH of the control calves between the 8 and 12 h time points. In human and equine medicine, achieving a gastric pH of >4 for >66% of a 24 h period is ideal for the healing of gastric ulcers (13). Our study's data suggest that both IV and SC administration of pantoprazole can achieve this therapeutic window, though more time points would be needed to confirm this timeframe. While these results are promising in calves, the efficacy of their use in adult ruminants is unclear due to vastly different gastrointestinal physiology. As calves are considered pre-ruminants, they function similarly to monogastric species because the esophageal groove allows milk to bypass the rudimentary rumen and enter directly into the abomasum (29, 30). Adult ruminants have a functional rumen, which is a constant source of ingesta that enters the abomasum. As food entering the abomasum is a stimulant for acid secretion, the near-constant movement of ingesta from the reticulo-rumen into the abomasum suggests a more prolonged period of acid production when compared to calves (31). Prolonged acid secretion may present a challenge for PPI therapy, and modifications may need to be made in dosing regimens.

The sulfone metabolite was detected in all calves after all administrations, with concentrations detectable at 24 and 36 h after day 1 and 3 dosing, respectively. This is similar to the tissue concentrations of pantoprazole sulfone detected in calves at 1–3 days after intravenous administration (19), but different from goats which had no detectable levels of pantoprazole sulfone after 4 h post intravenous administration at 1 mg/kg (23). While the sulfone metabolite is thought to be inactive, it is detectable for longer in tissues than the parent compound in calves (19), so future studies investigating tissue residue disposition could further determine the relationship between plasma and tissue pantoprazole sulfone levels.

**Figure 6:**
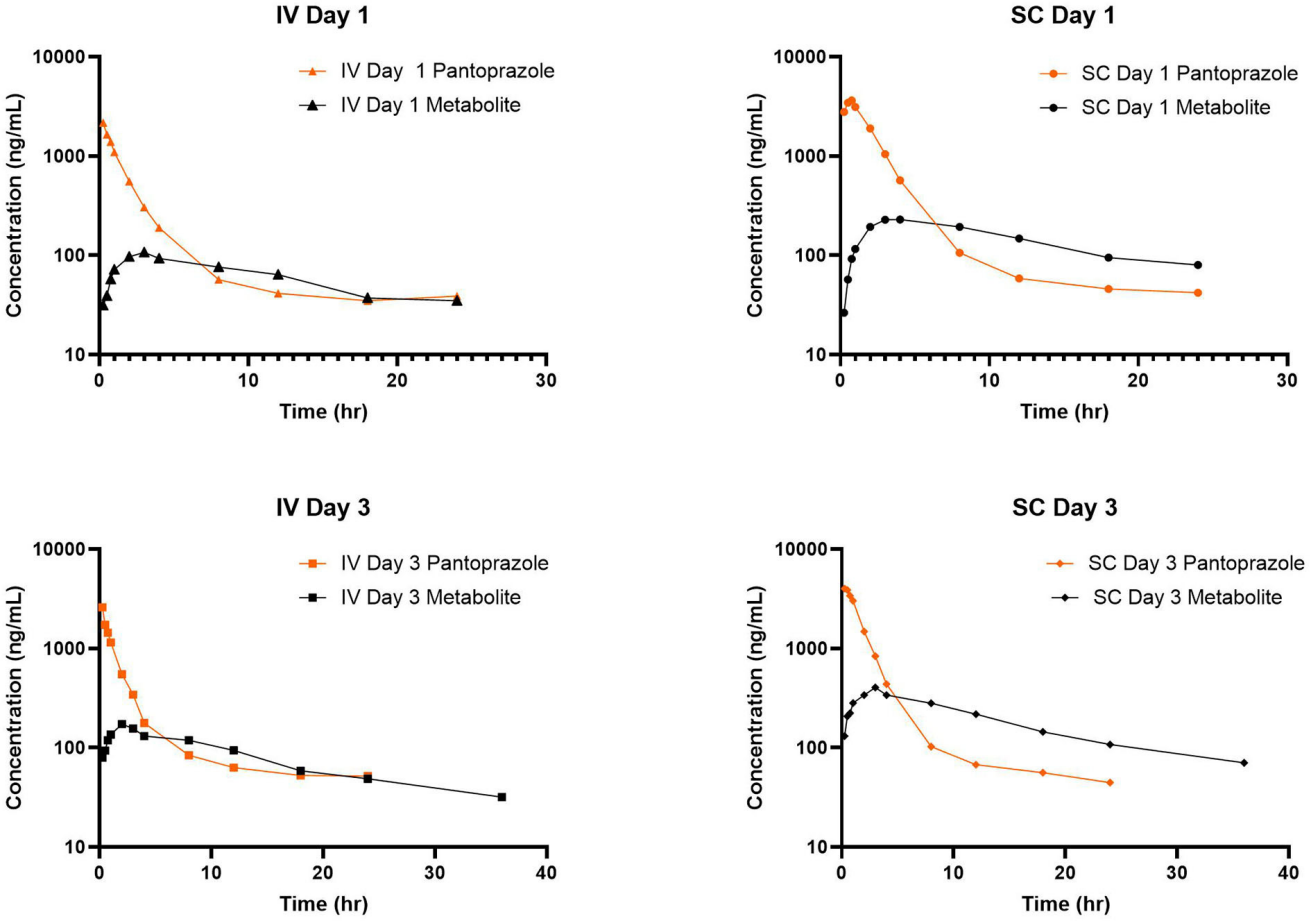
Comparison of time vs. concentration for parent and metabolite for each day and route of administration.

Arithmetic mean ± SD values of elimination rate constants (λz) for parent and sulfone metabolite were 0.47 ± 0.36 and 0.087 ± 0.024 1/h after intravenous administration on day 1; 0.38 and 0.08 1/h after subcutaneous administration on day 1; 0.15 ± 0.13 and 0.066 ± 0.03 1/h after intravenous administration on day 3; and 0.099 ± 0.094 and 0.54 ± 0.01 1/h after subcutaneous administration on day 3. Figure 6 displays the simultaneous time vs. concentration data for the parent and metabolite for each day and method of administration. From these values it appears that the formation of the sulfone metabolite is the rate limiting step in the pharmacokinetics of pantoprazole sulfone in calf. When evaluating the arithmetic mean ± SD λz for IV and SC parent on day one values are overall comparable at 0.47 ± 0.36 and 0.36 ± 0.22 1/h, respectively, while on day 3 these values are 0.15 ± 0.13 and 0.099 ± 0.094 1/h. Comparisons of λz for the metabolite after IV and SC administration are 0.087 ± 0.024 and 0.097 ± 0.053 1/h on day 1, and 0.066 ± 0.03 and 0.054 ± 0.01 1/h on day 3. While there are subtle differences between the slopes of pantoprazole after IV and SC administration, the similarities in elimination do not support flip flop pharmacokinetics for SC administration.

After the first SC administration, bioavailability (F) was noted to be 115%. While uncommon, a bioavailability of >100% can occur for several reasons, such as distribution between compartments, or as a result of sampling schedule. A bioavailability of >100% has been observed for pantoprazole in alpacas (115%) (14), as well as a similar proton pump inhibitor esomeprazole in goats (*F* = 116%) (32). This could indicate non-linear pharmacokinetics at higher dosages or potentially flip flop pharmacokinetics, although flip flop pharmacokinetics seems less likely comparing the slopes of each route.

All the calves tolerated both routes of administration well, and no pain or swelling was noted at the injection sites. Adverse events in humans include diarrhea, headache, and abdominal pain (33). All calves in the study maintained a healthy appetite and fecal consistency. Previous safety investigations of pantoprazole use in hospitalized ruminants also report similar findings (16).

The major limitation of this study is the small sample size that was used. While pharmacokinetic studies are appropriately powered with 4–6 animals, increasing the number of animals can decrease the impact of outlier data, as well as to highlight potential population variables which may describe the variation in some parameters, such as genetic polymorphisms. The animals in this study were also all from a single farm and all healthy, which is not reflective of the population of animals typically presenting to a hospital for care.

Future research should investigate the efficacy and safety of pantoprazole in sick populations, as well as investigate its use in adult ruminants. As this study has demonstrated that the abomasal pH can be increased with pantoprazole, it would be imperative to determine the effect on the microbiome of the gastrointestinal tract. Further investigations into the ideal dosage and frequency would also be important as therapy with pantoprazole can represent a substantial economic investment. As ruminants are part of the food supply and the use of pantoprazole is considered extra-label, establishing appropriate withdrawal times is vital. Additionally, while pH timepoints were collected at 18 hours, these were not presented, as it appears that additional sampling of pH after the second milk feeding should be considered for future studies in calves to truly account for pH changes from pantoprazole, vs. the second milk feeding. This model of cannulation for abomasal fluid sampling could be utilized for other gastroprotectant therapies in ruminants, such as esomeprazole, the S-enantiomer of omeprazole (32). Future studies could also evaluate the variation within individuals of a population to see if factors such as disease status or genetic polymorphism influence the metabolism of pantoprazole.

In conclusion, pantoprazole effectively increased abomasal pH in calves after either IV or SC administration, and multiple-day administration appeared to be well-tolerated. While further research is needed to determine the role of acid secretion in abomasal ulcer formation, this information is a step forward in ulcer management of ruminants.

## Data availability statement

The original contributions presented in the study are included in the article/supplementary material, further inquiries can be directed to the corresponding authors.

## Ethics statement

The animal study was reviewed and approved by Institutional Animal Care and Use Committee, University of Tennessee.

## Author contributions

JO, P-YM, LE, AK, JM, and JS devised the experimental design. SC and JB developed the analytical method. JO, P-YM, LE, JS, HC, CC, RR, and WS-G contributed to animal setup, husbandry, and data collection. JO, SC, JB, and HC contributed to sample analysis. JO, AK, JM, SC, and JS contributed to data interpretation. All authors contributed to manuscript development. All authors contributed to the article and approved the submitted version.
